# Mother's Own Milk and Bronchopulmonary Dysplasia: A Systematic Review and Meta-Analysis

**DOI:** 10.3389/fped.2019.00224

**Published:** 2019-06-06

**Authors:** Eduardo Villamor-Martínez, Maria Pierro, Giacomo Cavallaro, Fabio Mosca, Eduardo Villamor

**Affiliations:** ^1^Department of Pediatrics, School for Oncology and Developmental Biology (GROW), Maastricht University Medical Center (MUMC+), Maastricht, Netherlands; ^2^UOC TIN e Neonatologia, Dipartimento Salute Mamma e Bambino, Fondazione Poliambulanza, Brescia, Italy; ^3^Neonatal Intensive Care Unit, Department of Clinical Sciences and Community Health, Fondazione IRCCS Cà Granda Ospedale Maggiore Policlinico, Università degli Studi di Milano, Milan, Italy

**Keywords:** mother's own milk, human milk, bronchopulmonary dysplasia, preterm formula, meta-analysis, systematic review, meta-regression

## Abstract

**Background:** Bronchopulmonary dysplasia (BPD) is the most common complication of very preterm birth and can lead to lifelong health consequences. Optimal nutrition is a cornerstone in the prevention and treatment of BPD. In very preterm infants, mother's own milk (MOM) feeding is associated with lower risks of necrotizing enterocolitis, retinopathy of prematurity, and sepsis. Although several studies have shown that MOM may protect against BPD, a systematic analysis of the evidence has not been performed to date.

**Methods:** A comprehensive literature search was conducted using PubMed/MEDLINE and EMBASE, from their inception to 1 December 2017. Longitudinal studies comparing the incidence of BPD in preterm infants fed with exclusive MOM, MOM supplemented with preterm formula (PF), and/or exclusively fed with PF were selected. A random-effects model was used to calculate the Mantel Haenszel risk ratio (RR) and 95% confidence interval (CI).

**Results:** Fifteen studies met the inclusion criteria (4,984 infants, 1,416 BPD cases). Use of exclusive MOM feedings was associated with a significant reduction in the risk of BPD (RR 0.74, 95% CI 0.57–0.96, 5 studies). In contrast, meta-analysis could not demonstrate a significant effect on BPD risk when infants fed with more than 50% MOM were compared with infants fed with <50% MOM (RR 0.98, 95% CI 0.77–1.23, 10 studies) or when infants fed with MOM supplemented with PF were compared with infants fed with exclusive PF (RR 1.00, 95% CI 0.78–1.27, 6 studies). Meta-regression showed that differences in gestational age were a significant confounder of the effect of MOM.

**Conclusion:** To our knowledge, this is the first systematic review and meta-analysis that specifically evaluates the role of MOM on BPD. Our data indicate that MOM may reduce the incidence of BPD when used as an exclusive diet, but this result needs to be interpreted with caution. We did not find the same difference in analyses with other dosages of MOM. Further studies adequately powered to detect changes in BPD rates and that adjust for confounders are needed to confirm the beneficial effects of MOM on BPD.

## Introduction

Mother's own milk (MOM), fresh or frozen, is the normative standard for preterm infant feeding and nutrition (1–4). If MOM is unavailable despite significant lactation support, pasteurized donor human milk (DHM) is the recommended alternative over the use of bovine milk-based preterm formula (PF) (1–4). However, it is increasingly recognized that numerous MOM components which could contribute to its protective effects against adverse outcomes of prematurity are reduced or absent in DHM (5).

Bronchopulmonary dysplasia (BPD) is one of the most common complications of prematurity, and it predicts multiple adverse outcomes including chronic respiratory impairment and neurodevelopmental delay (6, 7). Optimal nutritional support is considered a cornerstone in the treatment/prevention of BPD (8). Recently, we performed a systematic review and meta-analysis on the effects of DHM on BPD (9). Meta-analysis of randomized controlled trials (RCTs) could not demonstrate that supplementation of MOM with DHM had a significant effect on BPD risk when compared to supplementation with PF. However, meta-analysis of observational studies showed a protective effect of DHM supplementation on BPD (9). Two very recent systematic reviews confirmed that the protective effects of human milk (i.e., MOM and/or DHM) on BPD are only observed in meta-analysis of observational studies (10, 11). Using the GRADE-system (12), the authors of these meta-analyses consider the evidence to be inconclusive.

Despite the important differences between DHM and MOM, the umbrella term “human milk” is frequently used to encompass both MOM and DHM, implying that the beneficial effects of MOM can be directly extrapolated to DHM (5, 13). Moreover, many of the studies and meta-analyses have compared PF feedings with various combinations of PF, MOM, and DHM. A recent meta-analysis evaluated the effects of MOM on retinopathy of prematurity (ROP) (14). This analysis excluded data on DHM and showed that the overall incidence of ROP was reduced among infants fed MOM compared with those fed PF. To the best of our knowledge, no systematic review has focused on the role of MOM in the development of BPD. The analysis of exclusive MOM vs. PF was beyond the scope of our previous study (9), and Miller et al. and Huang et al. did not study the effect of MOM separately from that of DHM (10, 11). Therefore, we aimed to conduct a systematic review and meta-analysis on the association between MOM/PF feeding and BPD development. The present meta-analysis does not include data on DHM.

## Methods

This study is a continuation of our previous review on DHM and BPD (9), and shares much of the same methodology. We expanded on the protocol of our earlier study, and specified the objectives, criteria for study inclusion, method for evaluating study quality, outcomes and statistical methodology. We report this study according to the guidelines of the Preferred Reporting Items for Systematic Reviews and Meta-Analysis (PRISMA) (15). The PRISMA checklist for this report can be found in the Supplementary Material.

### Data Sources and Search Strategy

We modified and expanded on the search strategy of our earlier review (9). We carried out a comprehensive literature search using PubMed/Medline and EMBASE, from their inception to March 1, 2018. The search strategy for PubMed used the following terms, including Mesh terms: (breast milk OR infant feeding OR mother's own milk) AND (preterm infant OR very low birth weight infant) AND (outcome OR bronchopulmonary dysplasia OR BPD) AND (observational study OR cohort study OR case-control). We used a similar strategy in EMBASE. We applied no language restrictions. We translated articles when needed. We included cohort and case-control studies in this review, as well as RCTs with an observational arm. Other types of studies were excluded, but when considered relevant, they were read to identify additional studies to include. We also used the “cited by” tool in Google Scholar and Web of Science to identify studies for inclusion. Moreover, we included articles which we came across in the elaboration of our earlier review (9).

### Eligibility Criteria and Study Selection

We included studies if they were original cohort or case-control studies, which examined very preterm (gestational age, GA <32 weeks) or very low birth weight (VLBW, BW <1,500 g) infants receiving either MOM or PF, and which included at least two groups divided according to feeding policy. Only full-length published studies were considered for inclusion. Studies were included if they reported results on the incidence of BPD. We defined BPD as oxygen dependence at 28 days of life (BPD28) or as oxygen dependence at 36 weeks adjusted gestational age (BPD36). Studies were excluded if the group receiving MOM or PF also received DHM. Two reviewers (EV-M, EV) independently screened the results of the searches, and included studies according to the inclusion criteria using EndNote (RRID:SCR_014001), using the methodology of Bramer et al. (16). Studies on which reviewers disagreed for inclusion were identified, and discrepancies were resolved through discussion or by consulting the other authors.

### Data Extraction

We collected the following information per study: citation information, study design, number of patients, number of centers, location of study, inclusion and exclusion criteria, patient characteristics (GA, BW), type of feeding received (MOM, PF, combination of MOM and PF, and type of fortifier), and incidence of BPD per group. Two researchers (EV-M, EV) extracted the data using an Excel sheet designed for this review. We resolved discrepancies in data extraction through discussion, or by consulting the other authors. Another researcher (MP) independently validated the accuracy of the data extracted.

### Assessment Risk of Bias

Two researchers (EV-M, MP) assessed the risk of bias in included studies. We used the Newcastle-Ottawa Scale (NOS) for quality assessment of cohort and case-control studies. The NOS is used to assign a score to studies on selection (0–4 points), comparability (0–2 points), and outcome/exposure (0–3 points), for a total score of up to 9 points. Discrepancies were resolved through discussion.

### Statistical Analysis

We used Comprehensive Meta-Analysis V3.0 software (RRID:SCR_012779) to combine and analyze studies. The Mantel Haenszel (MH) risk ratio (RR) for BPD with 95% confidence interval (CI) was calculated in each study. Due to anticipated heterogeneity, we used a random-effects model to combine studies. This model accounts for heterogeneity between and within studies and it does not assume that “true” effect sizes are identical across studies. Subgroup analyses were conducted according to the mixed-effects model (17). In this model a random-effects analysis is used to combine studies within each subgroup, and a fixed-effect model is used to combine subgroups and yield the overall effect. The model does not assume study-to-study variance (tau-squared) to be the same for all subgroups. We assessed statistical heterogeneity using the Cochran's Q statistic, and the *I*^2^ statistic which is derived from it. We planned to evaluate the risk of publication bias through visual inspection of the funnel plot and with Egger's regression test (18). We decided *a priori* to analyze the effect of GA as a confounding factor, by analyzing the mean difference in this covariate between groups, and through subgroup analysis, by removing studies with large differences in GA from analysis. We decided to use the group with the higher MOM intake as the reference group in all our analyses. We carried out sensitivity analyses by removing one study from analyses at a time. We used an α = 0.05 for statistical significance (α = 0.10 for statistical heterogeneity).

## Results

**Figure 1:**
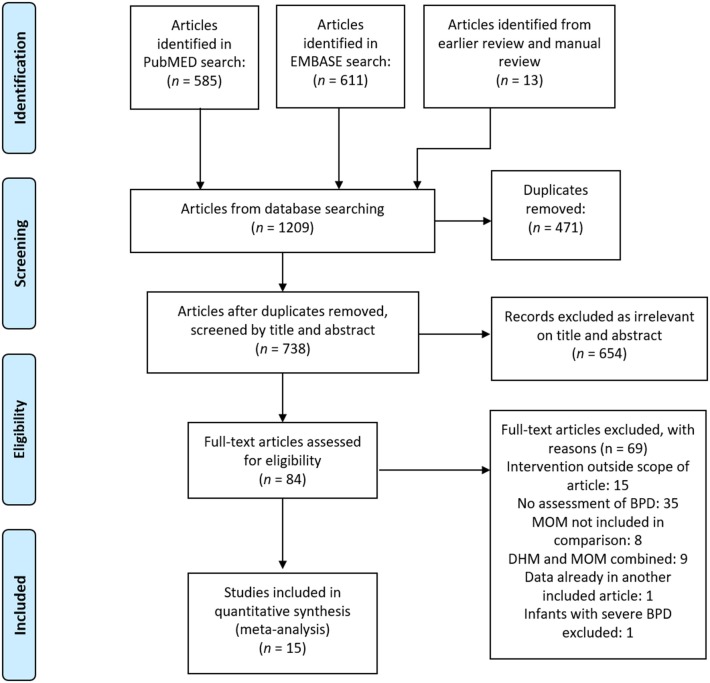
PRISMA diagram of the search.

After removing duplicates, our comprehensive search found 965 articles, of which we identified 84 as potentially relevant, and 15 met our inclusion criteria (19–33) after full-text review. The PRISMA search diagram is shown in Figure 1. The characteristics of the included studies are shown in Supplementary Table 1. Fourteen included studies were observational cohorts, of which seven were prospective (20, 21, 23, 25, 30, 31, 33) and seven were retrospective (19, 22, 24, 26, 28, 29, 32), and one study was a retrospective case-control (27). One study was excluded from meta-analysis because it did not group by type of feeding (22). We divided studies according to the proportion of feeding that was MOM or PF in each group, and we made three comparisons for analysis: (1) Exclusive MOM vs. Any PF; (2) Mainly MOM vs. Mainly PF; (3) Any MOM vs. Exclusive PF.

### Quality Assessment

Three studies scored six points on the NOS, 10 studies scored seven points, and two studies scored the maximum of 9 points. We downgraded studies in quality for not adjusting for confounders (*k* = 13), for excluding infants who were lost to follow-up (*k* = 2) and for not defining BPD clearly (*k* = 1).

### Exclusive MOM vs. Any PF

**Figure 2:**
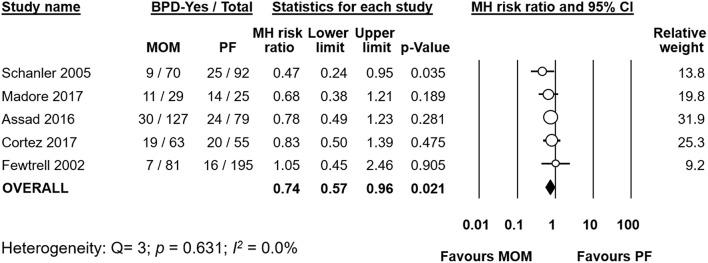
Meta-analysis of exclusive MOM vs. any PF and risk of BPD. MOM, mother's own milk; PF, preterm formula; BPD, bronchopulmonary dysplasia; CI, confidence interval; MH, Mantel-Haenszel.

Five studies (19–21, 27, 30) compared infants who received a diet of exclusive MOM to infants who received MOM and any supplementation with PF. Meta-analysis of these studies found that the exclusive MOM group had a reduced risk of BPD (RR 0.74, 95% CI 0.57–0.96, *p* = 0.021, Figure 2). When we excluded the study of Fewtrell et al. which used a different definition of BPD (BPD28), the effect of MOM on BPD remained significant (RR 0.71, 95% CI 0.54–0.93 *p* = 0.014). Sensitivity analysis showed that removing the study of Madore et al. (27) or the study of Schanler et al. (30) made the overall association no longer significant (Supplementary Figure 1).

When we analyzed the difference in GA between groups, meta-analysis did not find a significant difference (MD GA −0.06 weeks, 95% CI −0.38–0.25, *p* = 0.689, Supplementary Figure 2). None of the included studies had a mean difference in GA between groups which was larger than 0.3 weeks.

**Figure 3:**
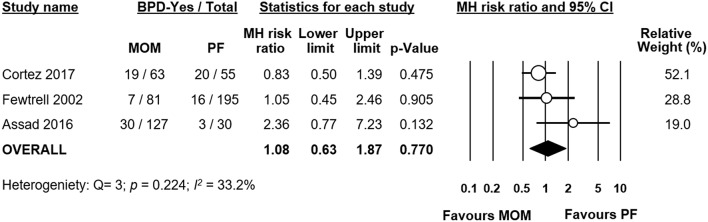
Meta-analysis of exclusive MOM vs. exclusive PF and risk of BPD. MOM, mother's own milk; PF, preterm formula; BPD, bronchopulmonary dysplasia; CI, confidence interval; MH, Mantel-Haenszel.

Out of the five studies which had an exclusive MOM group, three studies (19–21) also provided data on a group of infants receiving exclusive PF. We carried out a subgroup analysis of these studies. When pooled, meta-analysis could not find a significant difference in BPD risk between groups (RR 1.08, 95% CI 0.63–1.87, *p* = 0.770 Figure 3). When we excluded the study of Fewtrell et al., which defined BPD as BPD28 instead of BPD36, the effect of MOM did not change in significance (RR 1.10, 95% CI 0.58–2.09, *p* = 0.777). When we removed the study of Assad et al. for having a MD in GA (of 1.5 weeks) between groups ≥0.5 weeks, the results did not change in significance (RR 0.88, 95% CI 0.57–1.37, *p* = 0.583). An analysis on the difference in GA between groups did not find a significant difference overall (MD −0.41 weeks, 95% CI −1.19–0.36, *p* = 0.295, Supplementary Figure 3).

### Mainly MOM vs. Mainly PF

**Figure 4:**
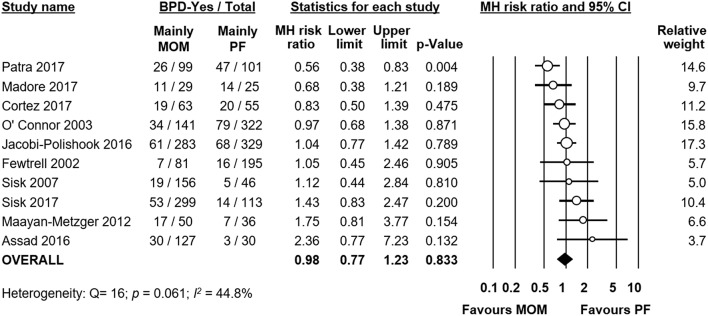
Meta-analysis of mainly MOM vs. mainly PF and risk of BPD. MOM, mother's own milk; PF, preterm formula; BPD, bronchopulmonary dysplasia; CI, confidence interval; MH, Mantel-Haenszel.

Ten studies compared infants receiving mainly MOM vs. infants receiving mainly PF. We included studies which had stricter criteria for comparison (i.e., exclusive MOM vs. exclusive PF) in this analysis as well. Meta-analysis could not find a significant difference in risk of BPD between the mainly MOM and the mainly PF group (RR 0.98, 95% CI 0.77–1.23, *p* = 0.833, Figure 4). When we excluded the study of O'Connor et al. for using a definition of BPD at 28 days of life instead of at 36 weeks PMA, the effect of MOM on BPD development remained non-significant (RR 0.99, 95% CI 0.75–1.31, *p* = 0.938). Excluding any study one at a time did not change the significance of the effect of MOM on BPD (Supplementary Figure 4).

We used meta-analysis to study the differences in GA between the MOM and PF groups in each study. Meta-analysis found no significant MD in GA when pooling all studies together (MD −0.31 weeks, 95% CI −0.78–0.17, *p* = 0.204, Supplementary Figure 5). However, individual studies showed significant differences in GA between groups, and the heterogeneity was very high (*p* < 0.001, *I*^2^ = 92.1%), which indicated that GA could be a significant confounder. When we used subgroup analysis to exclude studies where the difference in GA between groups was larger than 0.5 weeks, we were left with 6 studies, but the effect of MOM on BPD did not change significantly (RR 0.99, 95% CI 0.82–1.18, *p* = 0.890).

**Figure 5:**
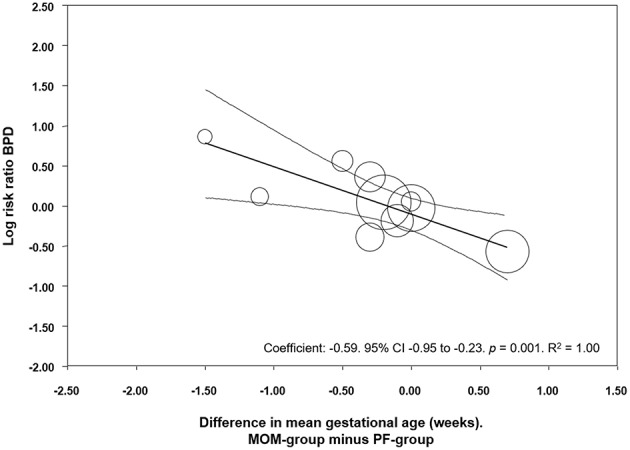
Meta-regression of MD in GA between the MOM and PF group, and risk of BPD. CI, confidence interval; BPD, bronchopulmonary dysplasia.

We used meta-regression to explore the role of GA in potentially modifying the effect of MOM on BPD development. Meta-regression found a significant association between MD in GA and the risk of BPD in the MOM group (Coefficient: −0.59, 95% CI −0.95 to −0.23, *p* = 0.001, *R*^2^ = 1.00, Figure 5). This indicates that in studies where the MOM group had a higher risk of BPD, this group was also more premature than the PF group, and in studies where the MOM group had a lower risk of BPD, this group was also more mature than the PF group.

### Any MOM vs. Exclusive PF

**Figure 6:**
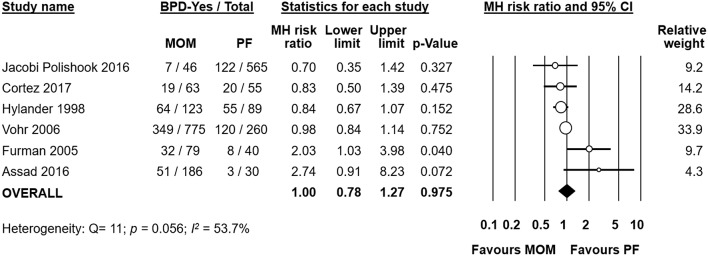
Meta-analysis of Any MOM vs. Exclusive PF and risk of BPD. MOM, mother's own milk; PF, preterm formula; BPD, bronchopulmonary dysplasia; CI, confidence interval; MH, Mantel-Haenszel.

Six studies (19, 20, 23–25, 33) compared infants who received any MOM to infants who received exclusive PF. We also included studies in this comparison where the infants of the MOM group received larger proportions of MOM (e.g., infants receiving mainly MOM or exclusive MOM). Meta-analysis could not find a significant effect of any MOM on the risk of developing BPD (RR 1.00, 95% CI 0.78–1.27, *p* = 0.975, Figure 6). When we removed the study of Hylander et al. from the analysis, which did not clarify their definition of BPD, the effect of any MOM remained non-significant (RR 1.07, 95% CI 0.80–1.42, *p* = 0.665). Removal of any one study did not affect the significance of the results (Supplementary Figure 6).

**Figure 7:**
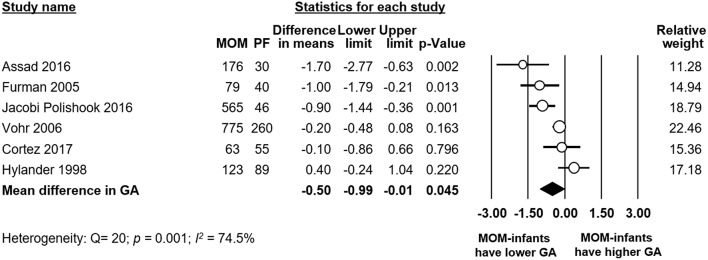
Meta-analysis of Any MOM vs. Exclusive PF, mean difference (MD) in gestational age (GA) between groups. MOM, mother's own milk; PF, preterm formula.

Meta-analysis found that there was a significant difference in mean GA between the any MOM group and the exclusive PF group, with the infants receiving any MOM being born earlier (MD −0.50 weeks, 95% CI −0.99 to −0.01, *p* = 0.045, Figure 7). Removing studies where the groups differed by more than 0.5 weeks in GA left us with three studies (20, 24, 33), but the effect of MOM on BPD remained non-significant (RR 0.93, 95% CI 0.82–1.05, *p* = 0.236, Supplementary Figure 7).

### Publication Bias

We tested the three comparisons for publication bias (Supplementary Figure 8), but neither visual inspection of the funnel plot nor Egger's regression test could find significant evidence of publication bias. A small number of studies made the analyses on “Exclusive MOM vs. Any PF” and “Any MOM vs. Exclusive PF” inconclusive (Supplementary Figure 8).

### Adjusted Data

Two studies (23, 26) reported data on BPD incidence that was adjusted for confounders. Furman et al. (23) reported incidence of BPD by amount of maternal milk received, and they adjusted for several confounders (BW, sex, and ethnicity). They found no significant difference in BPD risk for varying levels of MOM intake, compared to receiving exclusive PF. Maayan-Metzger et al. (26) used logistic regression to adjust for confounders including GA, BW and sex. They found that receiving only or mainly MOM, compared to receiving only or mainly PF, did not significantly affect the risk of developing BPD. They found the same result in the subgroup of infants with GA 24–28 weeks.

### Other Studies

One study (22) did not group according to MOM or PF intake. Instead they compared infants with BPD to infants without BPD and studied median intake of MOM in the first 6 weeks of life. In their study infants were given MOM, supplemented by PF when necessary. They found infants with BPD had a significantly lower median daily MOM intake compared to infants without BPD (2.3 mL/kg/d vs. 10.8). The protective effect on BPD of a higher MOM intake at 42 days remained after adjustment for confounding factors (RR 0.98, 95% CI 0.96–0.99, *p* = 0.030).

## Discussion

RCTs are widely regarded to provide the highest degree of evidence (34). However, the random allocation of infants to a group receiving PF instead of MOM is not ethical and, therefore, evidence must be based on observational studies (14, 35). To our knowledge, this is the first systematic review and meta-analysis that specifically evaluated the role of MOM on BPD. We found that MOM reduced the risk of developing BPD but only when used as an exclusive diet. In contrast, meta-analysis could not find significant changes in BPD risk when comparing infants fed mainly with MOM with those fed mainly with PF, or when comparing any MOM vs. exclusive PF.

The reduction of BPD rates when MOM is used as an exclusive diet may have various explanations. The major pathogenetic clue of BPD is the arrest in the alveologenesis and vasculogenesis of the lung due to very preterm birth (36). Superimposed inflammatory events complete this detrimental picture (37, 38). Prenatally, in the setting of chorioamnionitis, the overwhelming inflammatory cascade may interfere with lung development (37). Postnatally, the intensive care support needed by very preterm infants, including resuscitation, mechanical ventilation, and oxygen administration, carries a high grade of inflammation to the immature lung, leading to the establishment of BPD (38). When postnatal infections occur, the incidence of BPD sharply increases (39–41). Finally, inadequate nutrition can further worsen BPD (42). MOM may reduce the incidence of BPD thanks to nutritional and bioactive components, counteracting oxidative stress (43), inflammation (44, 45), and nutritional flaws involved in the BPD pathogenesis (46, 47). In addition, MOM may also impact the risk of BPD indirectly by reducing the incidence of necrotizing enterocolitis (NEC) and late-onset sepsis (LOS).

Due to the observational character of the studies included in the meta-analysis, the MOM and PF groups may differ in a number of maternal and infant characteristics which may affect the development of BPD. Previous studies have shown associations between characteristics such as ethnicity, socioeconomic status, maternal education, pregnancy hypertensive disorders, smoking during pregnancy, GA, BW, infant sex, Apgar score, or respiratory distress syndrome, and rate of MOM feeding in preterm infants (32, 48–54). We evaluated one possible major confounder: difference in GA between groups. GA played a role in modifying the association between MOM and BPD, as we have shown through meta-regression and sub-group analyses. This is relevant since the incidence of all the complications of prematurity, including NEC, LOS, and BPD, is inversely related to GA. In studies which compared mainly MOM vs. mainly PF, the comparison that included the highest number of studies, meta-regression showed a significant correlation between difference in GA and the protective effect of MOM on BPD (Figure 5). In other words, in the studies where the mainly MOM group had a higher BPD risk, this group was also more premature than the mainly PF group. Interestingly, in the comparison where we found a significant positive result (exclusive MOM vs. any PF), the differences in GA between groups were small. This suggests that the protective effects of exclusive MOM are not affected by GA as confounder in this analysis.

Several studies have reported that the effects of human milk in reducing the incidence of adverse outcomes of prematurity are dose-dependent (14, 23, 31, 55–58). It has been suggested that at least 50% of the infant's total enteral intake should be MOM in order to achieve a decreased incidence of NEC (13). With regards to BPD, Patel et al. have shown a 9.5% reduction in the risk of BPD for each 10% increase in MOM received from birth to 36 weeks PMA. This may generate a reduction in BPD risk up to 63% when an exclusive MOM diet is compared with an exclusive PF diet (55). Surprisingly, the present meta-analysis could not demonstrate a different rate of BPD in infants fed exclusive MOM when compared with infants fed exclusive PF. However, this analysis was based on only three studies (Figure 3). Moreover, in one of the studies the infants in the PF group had a markedly higher GA (1.5 weeks) than the infants of the MOM group. To date, there are no exact limits set in the amount of MOM that would produce benefits in terms of BPD reduction (59). The studies that we analyzed documented a high variability of MOM amount in their study groups. Since the relation between MOM and BPD, may not be as direct as for NEC and LOS, it is possible that higher minimum amounts of MOM may be needed to detect significant differences. In addition, the conditions of storage and the use of fresh, refrigerated, frozen, or deep-frozen MOM may affect the antioxidant as well as other biological properties of MOM (60).

## Conclusion

Our data indicate that MOM may reduce the incidence of BPD when used as an exclusive diet, but this result needs to be interpreted with caution. We did not find the same difference in analyses with other dosages of MOM, which may be related to the high variability in the available studies and the dose-dependent beneficial effects of MOM. It may also be due to differences in GA between infants who receive MOM and infants who receive PF, which we found had modified the protective effects of MOM against BPD. Moreover, there may be other differences in infant and maternal characteristics that play a role and which we could not account for. Further studies, adequately powered to detect changes in BPD rates, and that adjust for the different characteristics of infants who receive MOM and PF are needed to confirm the beneficial effects of MOM on BPD.

## Author Contributions

EV-M designed the study, performed the search, selected studies for inclusion, collected data, performed the statistical analyses, contributed to the interpretation of the results, and drafted the initial manuscript. MP revised collected data, contributed to statistical analysis and interpretation of the results, and reviewed and revised the manuscript. GC contributed to interpretation of results and reviewed and revised the manuscript. FM contributed to interpretation of results and reviewed and revised the manuscript. EV conceptualized and designed the study, performed the search, selected the studies for inclusion, supervised data collection, contributed to the statistical analyses and interpretation of the results, and reviewed and revised the manuscript. All authors approved the final manuscript as submitted.

### Conflict of Interest Statement

The authors declare that the research was conducted in the absence of any commercial or financial relationships that could be construed as a potential conflict of interest.
