# Supplementary material for: Mother's Own Milk and Bronchopulmonary Dysplasia: A Systematic Review and Meta-Analysis
Source: Front Pediatr. 2019 Jun 6;7:224. doi: 10.3389/fped.2019.00224 (PMC6593284; doi:10.3389/fped.2019.00224)
Supplement: Supplementary file 1 [file Data_Sheet_1.pdf]

Supplementary Material

Mother's Own Milk and Bronchopulmonary Dysplasia: A Systematic Review and Meta-Analysis

Eduardo Villamor-Martínez[1], Maria Pierro[2], Giacomo Cavallaro[3], Fabio Mosca[3], Eduardo Villamor[1*]

[1] Department of Pediatrics, Maastricht University Medical Center (MUMC+), School for Oncology and Developmental Biology (GROW), Maastricht, The Netherlands

[2] UOC TIN e Neonatologia, Dipartimento Salute Mamma e Bambino, Fondazione Poliambulanza Brescia, Italy

[3] Neonatal Intensive Care Unit, Department of Clinical Sciences and Community Health, Fondazione IRCCS Cà Granda Ospedale Maggiore Policlinico, Università degli Studi di Milano, Milan, 20122, Italy

*** Correspondence:**
Eduardo Villamor, MD PhD.
e.villamor@mumc.nl

1 Supplementary Figures and Tables

Study name	Point	Lower limit	Upper limit	p-Value
Cortez 2017	0.71	0.53	0.96	0.025
Fewtrell 2002	0.71	0.54	0.93	0.014
Assad 2016	0.72	0.53	0.99	0.041
Madore 2017	0.75	0.57	1.01	0.056
Schanler 2005	0.79	0.60	1.05	0.102
**Analysis with all studies**	**0.74**	**0.57**	**0.96**	**0.021**

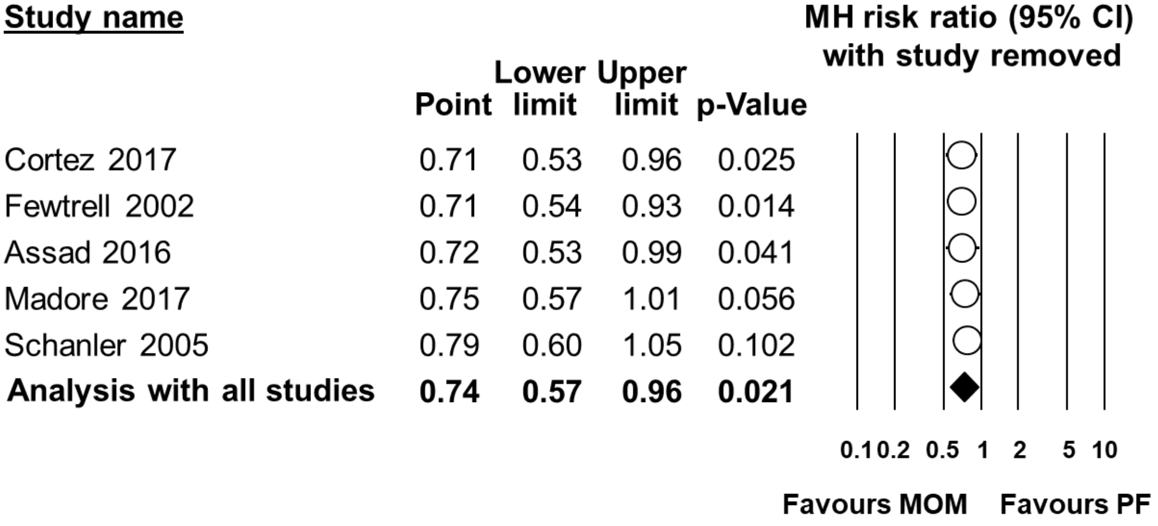

Supplementary Figure 1. Meta-analysis of Exclusive MOM vs. Any PF, effect of removing one study each time. MOM: mother's own milk; PF: preterm formula; MH: Mantel-Haenszel.

Study name	MOM	PF	Difference in means	Lower limit	Upper limit	p-Value	Relative weight
Madore 2017	29	25	-0.30	-1.26	0.66	0.542	10.47
Assad 2016	127	79	-0.10	-0.90	0.70	0.806	15.31
Cortez 2017	63	55	-0.10	-0.86	0.66	0.796	16.94
Fewtrell 2002	81	195	0.00	-0.55	0.55	1.000	32.11
Schanler	70	92	0.00	-0.62	0.62	1.000	25.17
**Mean difference in GA**			**-0.06**	**-0.38**	**0.25**	**0.689**	

Statistics for each study

Heterogeneity: Q= 3; p = 0.631; I^2 = 0.0%

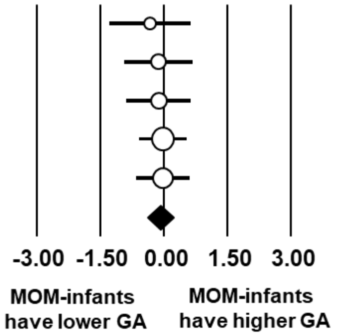

Supplementary Figure 2. Meta-analysis of Exclusive MOM vs. Any PF, mean difference (MD) in gestational age (GA) between groups. MOM: mother's own milk; PF: preterm formula.

Study name	MOM	PF	Difference in means	Lower limit	Upper limit	p-Value	Relative weight
Assad 2016	127	30	-1.50	-2.59	-0.41	0.007	25.25
Cortez 2017	63	55	-0.10	-0.86	0.66	0.796	34.22
Fewtrell 2002	81	195	0.00	-0.55	0.55	1.000	40.52
**Mean difference in GA**			**-0.41**	**-1.19**	**0.36**	**0.295**	

Statistics for each study

Heterogeneity: Q= 5.97; p = 0.051; I^2 = 66.5%

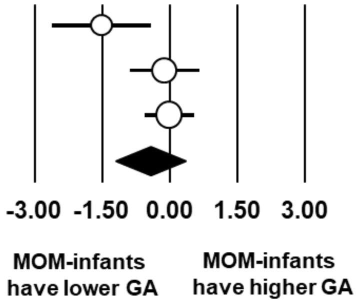

Supplementary Figure 3. Meta-analysis of Exclusive MOM vs. Exclusive PF, mean difference (MD) in gestational age (GA) between groups. MOM: mother's own milk; PF: preterm formula.

Study name	Point	Lower limit	Upper limit	p-Value
Sisk 2017	0.93	0.66	1.30	0.655
Maayan-Metzger 2012	0.93	0.67	1.28	0.642
Assad 2016	0.93	0.68	1.27	0.648
Sisk 2007	0.98	0.69	1.38	0.889
Jacobi-Polishook 2016	0.98	0.66	1.45	0.921
Fewtrell 2002	0.98	0.69	1.39	0.912
O' Connor 2003	1.00	0.68	1.46	0.982
Cortez 2017	1.02	0.72	1.44	0.930
Madore 2017	1.03	0.74	1.43	0.858
Patra 2017	1.09	0.87	1.37	0.460
**Analysis with all studies**	0.98	0.71	1.35	0.917

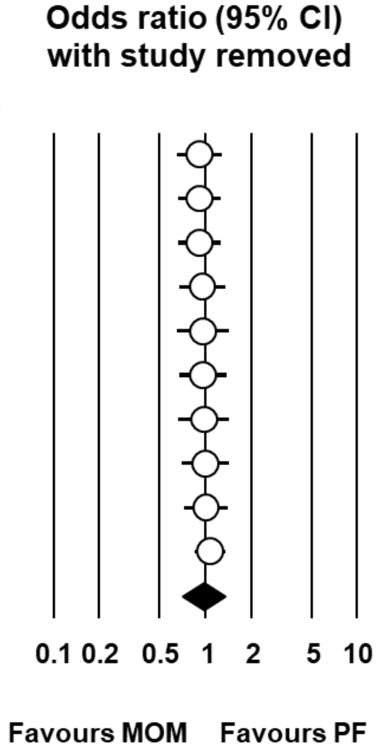

Supplementary Figure 4. Meta-analysis of Mainly MOM vs. Mainly PF, effect of removing one study each time. MOM: mother's own milk; PF: preterm formula.

Study name	MOM	PF	Difference in means	Lower limit	Upper limit	p-Value	Relative Weight (%)
			**Statistics for each study**				
Assad 2016	127	30	-1.50	-2.59	-0.41	0.007	7.51
Sisk 2007	156	46	-1.10	-1.17	-1.03	0.000	12.49
Maayan-Metzger 2012	50	36	-0.50	-1.65	0.65	0.396	7.17
Madore 2017	29	25	-0.30	-1.26	0.66	0.542	8.24
Sisk 2017	299	113	-0.30	-0.83	0.23	0.269	10.82
Jacobi-Polishook 2016	283	329	-0.20	-0.49	0.09	0.169	11.99
Cortez 2017	63	55	-0.10	-0.86	0.66	0.796	9.48
Fewtrell 2002	81	195	0.00	-0.55	0.55	1.000	10.71
O' Connor 2003	141	322	0.00	-0.39	0.39	1.000	11.55
Patra 2017	99	101	0.70	0.03	1.37	0.040	10.03
**Mean difference in GA**			**-0.31**	**-0.78**	**0.17**	**0.204**	

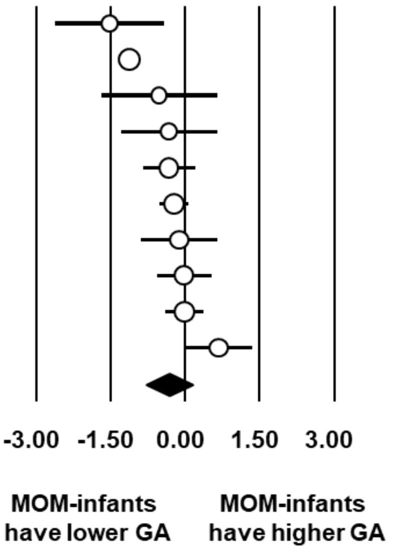

Heterogeneity: Q = 113; p <0.001; I^2 = 92.1%

Supplementary Figure 5. Meta-analysis of Meta-analysis of Mainly MOM vs. Mainly PF, mean difference (MD) in gestational age (GA) between groups. MOM: mother’s own milk; PF; preterm formula.

Study name	Statistics with study removed			
	Point	Lower limit	Upper limit	p-Value
Assad 2016	0.95	0.74	1.22	0.694
Furman 2005	0.92	0.71	1.19	0.538
Jacobi Polishook 2016	1.03	0.80	1.33	0.810
Vohr 2006	1.01	0.75	1.36	0.965
Cortez 2017	1.03	0.79	1.33	0.843
Hylander 1998	1.07	0.80	1.42	0.665
**Analysis with all studies**	**1.00**	**0.78**	**1.27**	**0.975**

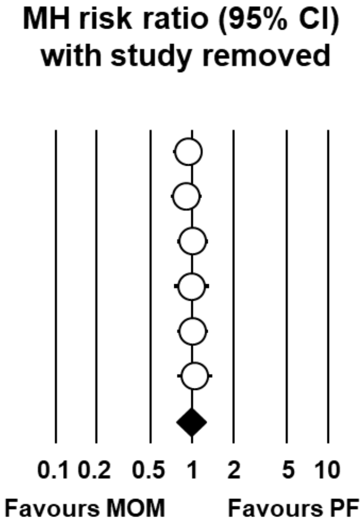

Supplementary Figure 6. Meta-analysis of Any MOM vs. Exclusive PF, effect of removing one study each time. MOM: mother’s own milk; PF: preterm formula.

Study name	BPD-Yes / Total		Statistics for each study				Relative weight
	MOM	PF	MH risk ratio	Lower limit	Upper limit	p-Value	
Cortez 2017	19 / 63	20 / 55	0.83	0.50	1.39	0.475	5.86
Hylander 1998	64 / 123	55 / 89	0.84	0.67	1.07	0.152	27.83
Vohr 2006	349 / 775	120 / 260	0.98	0.84	1.14	0.752	66.30
**Studies where MD GA <0.5 weeks**			**0.93**	**0.82**	**1.05**	**0.236**	

Heterogeneity: Q= 1; *p* = 0.533; I^2 = 0.%

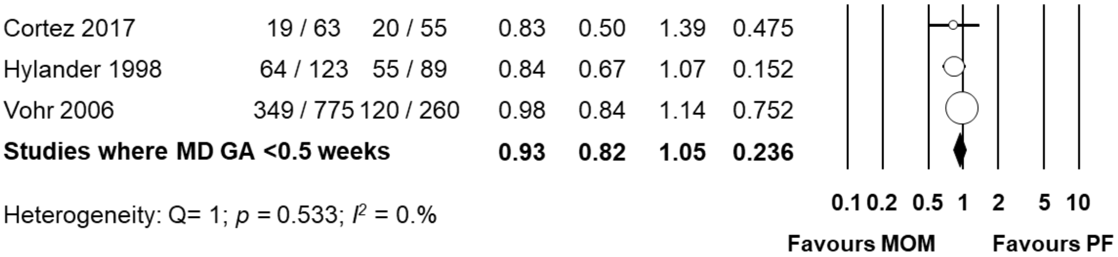

Supplementary Figure 7. Meta-analysis of Any MOM vs. Exclusive PF and risk of BPD, only including studies where the mean difference (MD) in gestational age (GA) was <0.50 weeks. MOM: mother’s own milk; PF: preterm formula; BPD: bronchopulmonary dysplasia; CI: confidence interval.

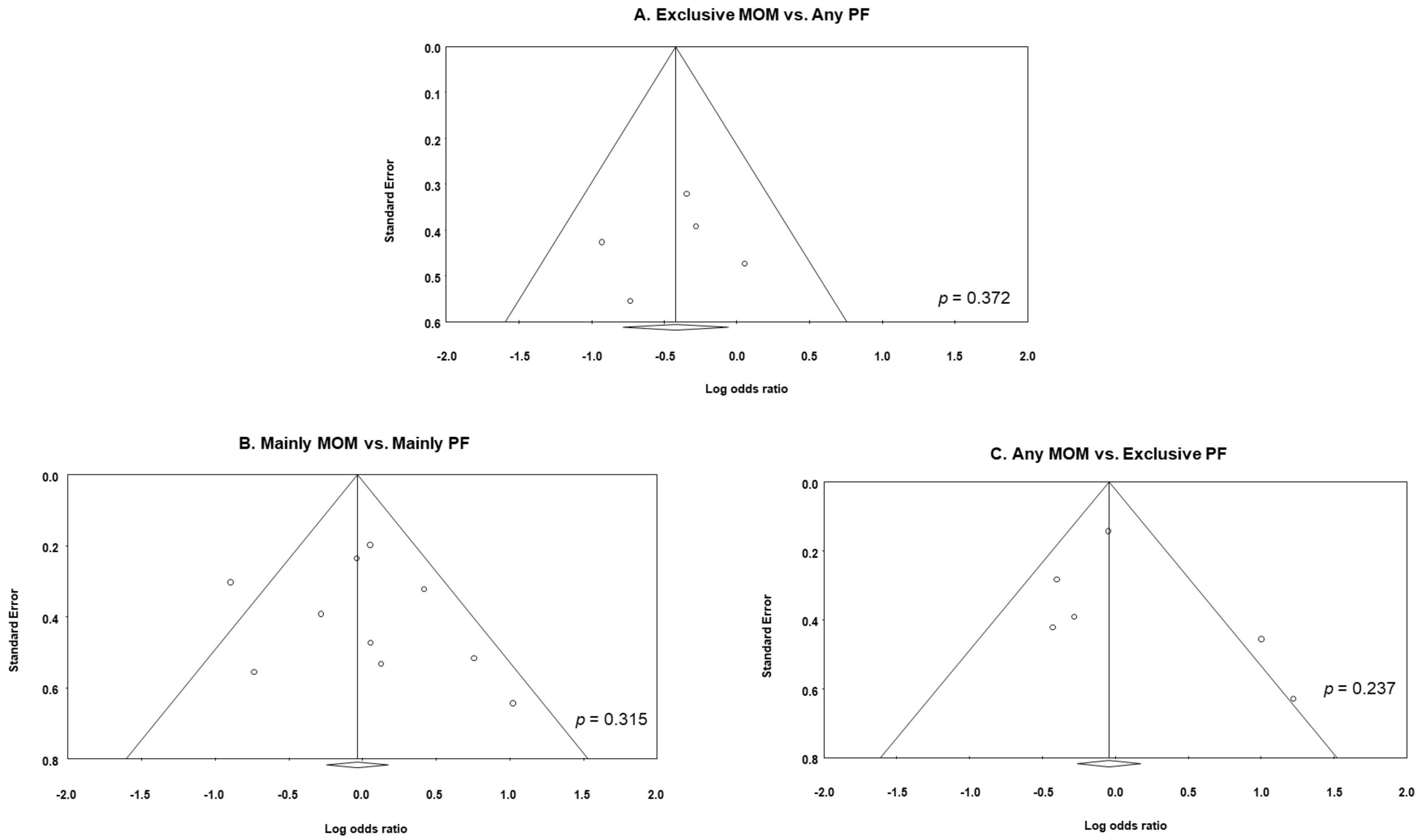

Supplementary Figure 8. Funnel plot analysis of publication bias. MOM: mother's own milk; PF: preterm formula.

Supplementary Table 1. Synoptic table of characteristics of all included studies.

First author, year	Location	Study design	Primary outcome(s)	Respiratory outcome(s)	Groups	Inclusion criteria	Study duration	Fortified	Patients (centers)	Comments
Assad et al. 2016	Baltimore, MD, USA	Retrospective cohort	Feeding intolerance, time to full feeds, NEC, length of stay, weight gain, cost of hospitalization	BPD36	- MOM + DHM + DHM-based fortifier;<br>- MOM + bovine fortifier;<br>- MOM + bovine fortifier + PF;<br>- Exclusive PF	GA <29 weeks and/or BW ≤1500g	Until discharge	DHM fortifier in exclusive human diet, bovine fortifier in bovine groups	293 (1)	
Cortez et al. 2017	Jacksonville, FL, USA	Prospective cohort	NEC, infection	BPD36	- Received >95% MOM<br>- Received >95% PF	GA <33 weeks	36h of age until discharge	Bovine fortifier added to MOM	118 (3)	
Fewtrell et al. 2002	London, UK	RCT (for our exposure: prospective cohort)	MDI and PDI at 18 months, Passamanick and Sherrard's Developmental Screening Inventory at 9 months PMA	BPD28	- Exclusive MOM<br>- Exclusive PF	BW <1750g, GA <37 weeks	Until discharge	Not specified	283 (3)	
Fonseca et al. 2017	Porto Alegre, Brazil	Retrospective cohort	Amount of MOM received by BPD patients vs. non-BPD infants	BPD28	MOM + PF in varying amounts, study compares MOM-intake by BPD patients vs. non-BPD patients	GA <32 weeks and/or BW <1500g	6 weeks or discharge	Bovine fortifier added to MOM	425 (1)	BPD was inversely associated with amount of MOM, even after controlling for confounders
Furman et al. 2003	Cleveland, OH, USA	Prospective cohort	Neonatal morbidity, length of hospitalization	BPD36	- Exclusive PF<br>- 1-24 mL/kg/d MOM + PF<br>- 25-49 mL/kg/d MOM + PF<br>- ≥50 mL/kg/d MOM + PF	GA <33 weeks, BW <1500g	4 weeks	Bovine fortifier added to MOM	119 (1)	MOM-intake divided over 4 groups by volume of MOM (in mL/kg/d), 0, 1-24, 25-49 and ≥50.
Hylander et al. 1998	USA	Retrospective cohort	Infection (culture proven sepsis, NEC and/or pneumonia)	BPD?	- Any MOM<br>- Exclusive PF	BW <1500g.	Until discharge	Bovine fortifier added to MOM	212 (1)	
Jacobi-Polishook et al. 2016	Boston, MA, USA	Prospective cohort	Neurodevelopmental outcome	BPD36	- Exclusive PF	GA ≤33 weeks	40 weeks corrected age	Bovine fortifier added to MOM	611 (5)	

First author, year	Location	Study design	Primary outcome(s)	Respiratory outcome(s)	Groups	Inclusion criteria	Study duration	Fortified	Patients (centers)	Comments
			(Bayley II) at 18 months		- MOM + PF, divided into four quartiles based on MOM-intake					
Maayan-Metzger et al. 2012	Tel Aviv, Israel	Retrospective cohort	Short-term neonatal outcomes	BPD28	- Only and mainly MOM<br>- Only and mainly PF	GA ≤32 weeks	Until discharge	Bovine fortifier added to MOM	360 (1)	Data taken from large prospective, randomized controlled trial, designed to assess possible benefits of supplementing formula with arachidonic and docosahexeanoic acid
Madore et al. 2017	Boston, MA, USA	Retrospective case-control	Growth, neurodevelopment	BPD36	- Exclusive MOM;<br>- PF>50%	BW <1000g	First month of life	Bovine fortifier added to MOM	81 (1)	
O' Connor et al. 2003	Toronto, Canada	Retrospective cohort	Growth and development outcomes	BPD28	- >80% MOM + PF<br>- ≥50% MOM + PF<br>- <50% MOM +PF<br>- MOM + >80% PF	GA <33 weeks	Until term corrected GA.	Bovine fortifier added to MOM	463 (9)	
Patra et al. 2017	Chicago, IL, USA	Retrospective cohort	Neurodevelopmental outcome	BPD36	MOM + PF, split into 5 quintiles based on proportion of MOM as total intake	GA <35 weeks, BW <1500g	Until 20 months corrected GA	Bovine fortifier added to MOM	251 (1)	Study uses same sample as Patel et al. 2017. We used the data from this article for meta-analysis.
Schanler et al. 2005	Houston, TX, USA	RCT (for our exposure: prospective cohort)	Late onset sepsis and/or NEC	BPD36	- Exclusive MOM<br>- MOM + PF	GA ≤29 weeks	19 days or discharge	Bovine fortifier added to MOM	243 (1)	
Sisk et al. 2007	Winston-Salem, NC, USA	Prospective cohort	NEC	BPD36	- MOM >50%<br>- PF >50%	BW 700-1500g	Until discharge	Bovine fortifier added to MOM	202 (1)	
Sisk et al. 2017	Winston-Salem, NC, USA	Retrospective cohort	NEC stage ≥2	BPD36	- MOM ≥50%<br>- DHM ≥50%<br>- PF ≥50%	GA ≤32w and BW ≤1500g	Within 2 hours of birth until 34 weeks PMA	Bovine fortifier added to MOM and to DHM	563 (1)	
Vohr et al. 2006	15 centers, USA	Prospective cohort	Neurodevelopmental outcome at 18 months	BPD36	- Any MOM + PF<br>- Exclusive PF	BW ≤1000g	Until discharge	Bovine fortifier added to MOM (varied per center)	1035 (15)	

BPD28: Defined as supplemental oxygen after day 28 of life. BPD36: Defined as supplemental oxygen at 36 weeks corrected GA. BPD28-36: defined as supplemental oxygen after day 28 of life, or at 36 weeks corrected GA age. BPD?: No definition of bronchopulmonary dysplasia given. MOM: Mother's own milk. PF: Preterm formula. DHM: Donor human milk

PRISMA 2009 Checklist

Section/topic	#	Checklist item	Reported on page #
**TITLE**			
Title	1	Identify the report as a systematic review, meta-analysis, or both.	1
**ABSTRACT**			
Structured summary	2	Provide a structured summary including, as applicable: background; objectives; data sources; study eligibility criteria, participants, and interventions; study appraisal and synthesis methods; results; limitations; conclusions and implications of key findings; systematic review registration number.	1
**INTRODUCTION**			
Rationale	3	Describe the rationale for the review in the context of what is already known.	2
Objectives	4	Provide an explicit statement of questions being addressed with reference to participants, interventions, comparisons, outcomes, and study design (PICOS).	2
**METHODS**			
Protocol and registration	5	Indicate if a review protocol exists, if and where it can be accessed (e.g., Web address), and, if available, provide registration information including registration number.	2,3
Eligibility criteria	6	Specify study characteristics (e.g., PICOS, length of follow-up) and report characteristics (e.g., years considered, language, publication status) used as criteria for eligibility, giving rationale.	3
Information sources	7	Describe all information sources (e.g., databases with dates of coverage, contact with study authors to identify additional studies) in the search and date last searched.	3
Search	8	Present full electronic search strategy for at least one database, including any limits used, such that it could be repeated.	3
Study selection	9	State the process for selecting studies (i.e., screening, eligibility, included in systematic review, and, if applicable, included in the meta-analysis).	3
Data collection process	10	Describe method of data extraction from reports (e.g., piloted forms, independently, in duplicate) and any processes for obtaining and confirming data from investigators.	3
Data items	11	List and define all variables for which data were sought (e.g., PICOS, funding sources) and any assumptions and simplifications made.	3
Risk of bias in individual studies	12	Describe methods used for assessing risk of bias of individual studies (including specification of whether this was done at the study or outcome level), and how this information is to be used in any data synthesis.	3
Summary measures	13	State the principal summary measures (e.g., risk ratio, difference in means).	4
Synthesis of results	14	Describe the methods of handling data and combining results of studies, if done, including measures of consistency (e.g., I^2) for each meta-analysis.	4

PRISMA 2009 Checklist

Section/topic	#	Checklist item	Reported on page #
Risk of bias across studies	15	Specify any assessment of risk of bias that may affect the cumulative evidence (e.g., publication bias, selective reporting within studies).	4
Additional analyses	16	Describe methods of additional analyses (e.g., sensitivity or subgroup analyses, meta-regression), if done, indicating which were pre-specified.	4
**RESULTS**			
Study selection	17	Give numbers of studies screened, assessed for eligibility, and included in the review, with reasons for exclusions at each stage, ideally with a flow diagram.	4
Study characteristics	18	For each study, present characteristics for which data were extracted (e.g., study size, PICOS, follow-up period) and provide the citations.	4, Supplementary Table 1
Risk of bias within studies	19	Present data on risk of bias of each study and, if available, any outcome level assessment (see item 12).	4
Results of individual studies	20	For all outcomes considered (benefits or harms), present, for each study: (a) simple summary data for each intervention group (b) effect estimates and confidence intervals, ideally with a forest plot.	4-7
Synthesis of results	21	Present results of each meta-analysis done, including confidence intervals and measures of consistency.	4-7, Supplementary Figures
Risk of bias across studies	22	Present results of any assessment of risk of bias across studies (see Item 15).	7
Additional analysis	23	Give results of additional analyses, if done (e.g., sensitivity or subgroup analyses, meta-regression [see Item 16]).	4-7
**DISCUSSION**			
Summary of evidence	24	Summarize the main findings including the strength of evidence for each main outcome; consider their relevance to key groups (e.g., healthcare providers, users, and policy makers).	7
Limitations	25	Discuss limitations at study and outcome level (e.g., risk of bias), and at review-level (e.g., incomplete retrieval of identified research, reporting bias).	8
Conclusions	26	Provide a general interpretation of the results in the context of other evidence, and implications for future research.	8
**FUNDING**			
Funding	27	Describe sources of funding for the systematic review and other support (e.g., supply of data); role of funders for the systematic review.	9

From: Moher D, Liberati A, Tetzlaff J, Altman DG, The PRISMA Group (2009). Preferred Reporting Items for Systematic Reviews and Meta-Analyses: The PRISMA Statement. PLoS Med 6(7): e1000097. doi:10.1371/journal.pmed1000097

For more information, visit: **www.prisma-statement.org**.
